# Efficiency of genomic and phenomic selection using mid-infrared milk spectra for milk production, somatic cell count, and udder type traits in French Lacaune dairy sheep

**DOI:** 10.3168/jdsc.2024-0714

**Published:** 2025-04-28

**Authors:** C. Machefert, H. Larroque, J.M. Astruc, C. Robert-Granié

**Affiliations:** 1 GenPhySE, Université de Toulouse, INRAE, ENVT, F-31326, Castanet-Tolosan, France; 2 Institut de l'Elevage, F-75595 Paris, France

## Abstract

Genomic selection uses molecular and pedigree information to accurately estimate genomic breeding values of animals from birth for traits in selection. Recent research in phenomic selection in plant production is opening up new opportunities in animal breeding. The approach of phenomic selection has been little studied in animal production. Here, we evaluate the efficiency of phenomic selection to estimate the phenomic values of phenotype-free females using mid-infrared spectral (MIRS) data from their milk samples. The phenotypes of 1,531 first-lactation French Lacaune dairy ewes were considered for traits included classically in the breeding goals, such as milk production and functional traits (SCS and udder type traits). The inclusion of standardized raw MIRS data instead of SNPs led to very low phenomic predictive abilities for udder type traits (Pearson correlations between phenotype and phenomic values from −0.08 to 0.07). For milk production traits, the phenomic predictions were superior to the genomic ones, in particular for lactation SCS (LSCS), with a predictive ability at 0.49 instead of 0.04. Overall, random regression-BLUP and Bayesian reproducing kernel Hilbert space methods gave equivalent results on phenomic predictions across all traits, with no impact from spectral data preprocessing. Finally, the efficiency of the combination of SNPs and milk MIRS in prediction models was low (average +3.8% for milk production and LSCS traits). Phenomic predictions could open up new prospects especially for the selection of nongenotyped females.

## Highlights

• Phenomic predictions with milk MIRS were higher than genomic predictions for milk production traits.
• Averaging lactation MIRS improved phenomic predictions over using a single spectrum.
• Random regression-best linear unbiased prediction and Bayesian reproducing kernel Hilbert space methods provided similar phenomic prediction accuracies.
• Milk MIRS data preprocessing methods have no impact on phenomic predictions.
• Adding milk MIRS to single nucleotide polymorphisms (SNPs) in prediction models did not improve phenotype predictions.

## Graphical Abstract

**Figure:**
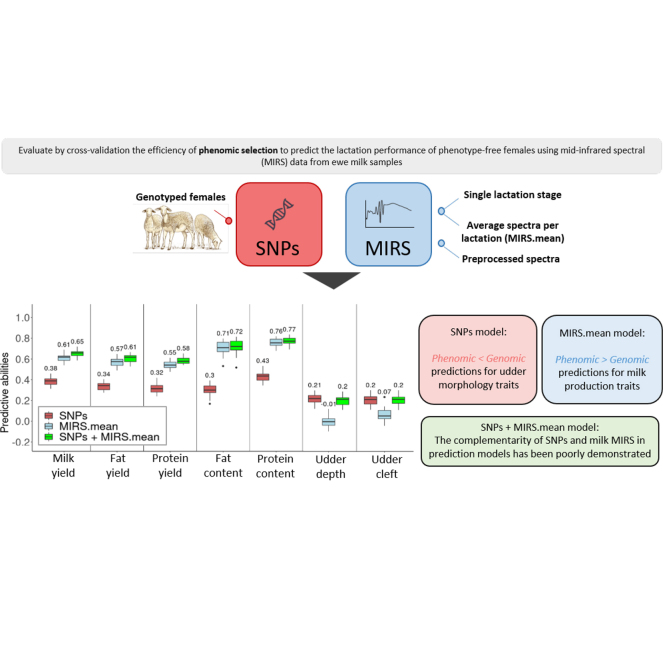
**Summary:** Animal breeding programs traditionally use phenotype and pedigree information to estimate breeding values of candidates to select for the traits to be improved. In dairy sheep, phenotypes are collected from ewes on-farm, and milk quality (protein and fat content) is measured from milk samples analyzed by midinfrared spectroscopy. Since 2015, the French Lacaune breed has switched to genomic selection, additionally using molecular information from genotypes to increase the accuracy of breeding values. This study tested a new approach called phenomic selection, using milk mid-infrared spectra (MIRS) instead of the molecular information to predict phenotypes used in national genomic evaluations. The results showed that using MIRS enabled more accurate prediction of phenotypes for milk production and udder health traits compared with molecular markers. However, this was not the case for udder type traits. The simultaneous use of molecular markers and MIRS data did not improve the phenotype predictions.

Genomic selection has been adopted by the breeding schemes to improve accuracy of the estimated breeding values for traits in selection from birth (Hayes et al., 2009). The single-step GBLUP model, which integrated all performance, pedigree, and genotypic information, significantly improved prediction accuracy (Legarra et al., 2014). In small dairy ruminants, this model outperformed the previous one based on performance and pedigree data only (Baloche et al., 2014; Carillier et al., 2014) and was adopted for the official French genetic evaluation in 2015 in the Lacaune sheep breed. The main advantage of using genomic information is the greater accuracy of estimated breeding values for candidates at birth. In small ruminants, evaluation of dairy traits is based on female phenotypes, whereas genotyping is mainly carried out on males due to its relative high cost. To be used for breeding purposes, phenotypic measures must accurately capture biological mechanisms, be heritable, and permit early and cost-effective measurement across large animal populations (De Marchi et al., 2014; Brito et al., 2021). Mid-infrared spectra (**MIRS**) of milk are high-throughput, low-cost measurements and routinely predict the major milk components (fat, protein, lactose) used in national genomic evaluations in dairy species (De Marchi et al., 2014), as well as finer compositions (Cesarani et al., 2019), cheese-making abilities (Sanchez et al., 2022), and other milk-related phenotypes (Rojas de Oliveira et al., 2024). The variability of transmittance at each wavenumber of milk MIRS is partly of genetic origin, showing very low heritability in water absorption areas to high heritability (>0.40) in specific spectral zones of milk components, regardless of the species (Soyeurt et al., 2010; Dagnachew et al., 2013; Machefert et al., 2024). Given that spectral data can capture genetic information, phenomic selection has been suggested as an alternative to genomic selection by switching markers to spectra data for inferring relationships between individuals in plant breeding (Rincent et al., 2018). These authors demonstrated that near-infrared spectral (**NIRS**) data, with heritabilities from 0 to 0.6, captured genetic similarities and provided phenomic predictions as accurate as, or even surpassing, genomic predictions for traits unrelated to the measured wheat and poplar tissues on which the spectrum was measured. Phenomic predictions based on NIRS have been reported for various crops, including grapevines (Brault et al., 2022), wheat (Jackson et al., 2023; Dallinger et al., 2023), and maize (Lane et al., 2020). Phenomic predictions were generally derived from simple and efficient models for estimating genetic effects, called hyperspectral BLUP (**HBLUP**), similar to GBLUP or ridge regression BLUP (**RR-BLUP**; Robert et al., 2022), or Bayesian models that account for varying marker effects (Meuwissen et al., 2001). However, integrating phenomic data like NIRS in evaluations may enhance predictions by accounting for nonadditive genetic effects, capturing environmental variation, and complementing genomic markers for genotype performance across environments (Robert et al., 2022; Brault et al., 2022; Jackson et al., 2023). The use of spectra in genetic models to capture genetic and environmental signals represents an innovative application of spectral data compared with their usual purpose for direct phenotype prediction via conventional methods such as partial least squares regression. In animal production, the approach of phenomic selection has been little studied. Jaouahdou et al. (2022) tested its feasibility in rainbow trout by comparing breeding value predictions using pedigree, genetic markers, and Raman spectral data from visceral adipose tissue. Phenomic models provided the best predictions for traits measured by Raman spectrometry and body weight, but for other growth traits not measured by spectrometry, predictions were less accurate than with genomic models. Recently, Odah et al. (2024) showed in French dairy cattle that phenomic prediction accuracy using MIRS of milk averaged 73% of that obtained with genomic models for milk production traits, 89% for functional traits (udder health, fertility), and 42% for morphological traits (height at sacrum).

From the original data provided by the European H2020 SMARTER project (2018–2023), the effectiveness of phenomic selection using milk MIRS was tested in dairy sheep. The study focused on traits under genomic selection in the French Lacaune dairy sheep breed, including milk production (milk yield, fat and protein yields, and contents) and functional traits (SCS and udder type traits).

This study was exempt from formal institutional animal care and use approval because data were recorded according to normal practices (agronomic or veterinary practice) in commercial farms in France. The dataset consisted of 1,531 first-lactation French Lacaune dairy ewes from 8 commercial farms located in southern France. The females came from 225 sires with at least 3 daughters, and from 1,212 dams. The data were collected during 2 milk production years from September 2019 to September 2021. Milk samples were collected during 6 monthly test days from the second to seventh month of lactation, starting 1 month after lambing when suckling ended. Lactation stage was defined the time gap between lambing and test-day in months. Milk spectral data were obtained using MilkoScan FT+ analyzers (FOSS, Hillerød, Denmark) with Fourier-transform infrared (**FTIR**) spectrometry at Agrolab's laboratory (France). Each sample's MIRS included 1,060 spectral points (wavenumbers) mainly in mid-infrared spectral region between 5,012 and 926 cm^−1^ and were expressed in transmittance. The spectra were standardized using a piece direct standardization method to reduce variability over time and between analyzers (Wang et al., 1991; Grelet et al., 2015). From the standardized raw spectra (**raw**), 6 preprocessing methods were applied: standard normal variate (**norm**) for centering and scaling, detrend (**dt**) for removing baseline (Barnes et al., 1989), first and second derivative on raw spectra (**der1** and **der2**, respectively) using the Savitzky and Golay (1964) procedure for removing baseline and enhance specific signals, and first and second derivative on normalized spectra (**norm_der1** and **norm_der2**, respectively). Following manufacturer recommendations, 3 spectral regions (5,012–2,975 cm^−1^, 2,431–2,276 cm^−1^, and 1,713–1,547 cm^−1^) were omitted due to water absorption noise or lack of relevant chemical bonds (FOSS, 1998). The final analysis included 446 selected wavenumbers, as in Machefert et al. (2024). All the traits considered were those included in the Lacaune breeding goals. The phenotypic data used were obtained by adjusting the ewes' lactation performance for fixed and nongenetic random effects extracted from GBLUP model of the routine genomic evaluations. Five milk production traits were analyzed for the whole lactation: milk yield (**MY**), fat content (**FC**), protein content (**PC**), fat yield (**FY**), and protein yield (**PY**). Fat content and PC were classically predicted by FTIR spectrometry, and FY and PY were obtained by the multiplication of MY by FC and PC, respectively. Lactation SCS (**LSCS**) was the average of SCS adjusted for stage of lactation for the first 3 test days at the morning milking (Barillet, 2007). Four udder morphological traits were phenotyped once per ewe (Marie-Etancelin et al., 2005): teat angle (**TA**), teat position anteroposterior (**TP-AP**), udder depth (**UD**), and udder cleft (**UC**). These traits were not related to milk quality and therefore to milk MIRS, and could be considered as control traits. The 1,531 ewes were genotyped with the Illumina Sheep LD consortium array (Illumina Inc., San Diego, CA), then imputed to obtain 38,523 SNPs that were retained for genomic evaluation of the French Lacaune dairy sheep breed. The SNPs with a minor allele frequency below 1%, call rate under 97%, or monomorphic markers were excluded. Hardy–Weinberg equilibrium was tested, and SNPs with a *P*-value below 1.10^−6^ were removed, as in Teissier et al. (2019).

The predictive abilities of 3 models to predict the 10 adjusted phenotypes were compared: genomic predictions based on SNP markers, phenomic predictions based solely on milk MIRS, or predictions summing the phenomic and genomic predictions obtained from a model including both effects and therefore based on both SNP and MIRS information. Two methods were tested: RR-BLUP and Bayesian reproducing kernel Hilbert spaces (**RKHS**) using 10,000 iterations with a burn-in of 2,000 iterations, run with the R (R Core Team, version 4.3.1) packages rrBLUP (Endelman, 2011) and BGLR (Pérez and de los Campos, 2014), respectively. Genomic predictions were obtained from the following model: **y** = **1***_n_μ* + **g** + *e*, where **y** is the vector of adjusted phenotypes, **1***_n_* is a vector of ones with a fixed intercept *μ*, **g** is the random effects of each genotype **g***_i_* with distribution $g∼N\left(0,G\sigma_{g}^{2}\right)$ and the genetic variance $\sigma_{g}^{2},$ and *e* is the random errors assumed to follow $e∼N\left(0,I\sigma_{e}^{2}\right),$ with **I** being the identity relationship matrix and $\sigma_{e}^{2}$ the error variance. Phenomic predictions were achieved using the HBLUP model: **y** = **1***_n_μ* + **h** + *e*, where **h** is a vector of random phenomic features with distribution $h∼N\left(0,H\sigma_{h}^{2}\right)$ and the phenomic variance $\sigma_{h}^{2}.$ Finally, SNP and MIR spectra information were integrated into a single prediction model: **y** = **1***_n_μ* + **g** + **h** + *e*. The genomic relationship matrix (**G**) was defined by VanRaden (2008), as follows:

$$G=\frac{ZZ^{\prime }}{2\Sigma p_{i}\left(1-p_{i}\right)},$$

where **Z** is a centered matrix of SNP genotypes and *p_i_* is the estimated allele frequency at locus *i*. The phenomic (hyper)spectral relationship matrix (**H**) was defined by Robert et al. (2022), as follows:

$$H=\frac{SS^{\prime }}{n_{w}},$$

where **S** is a centered and scaled matrix for each wavenumber and *n_w_* is the number of wavenumbers (*n_w_* = 446). Three types of spectra were considered: average spectra (**MIRS.mean**) made from an average of 3 MIRS spectra, and the spectrum at lactation stage 2 (**MIRS.stage2**) and stage 6 (**MIRS.stage6**). The MIRS.mean represented overall lactation performance, MIRS.stage2 evaluated predictive ability at the start of lactation, and MIRS.stage6 captured, in addition to the end-of-lactation effect, the impact of dietary changes, such as the transition to pasture typically observed on French dairy sheep farms.

The efficiency of genomic and phenomic predictions was assessed through 25 cross-validations, using 80% of the dataset for training and 20% for validation (20% of the ewes from each farm). In the validation set, adjusted phenotypes of ewes were omitted. Predictive ability was computed as the Pearson correlation between predictions values (genomic or phenomic) and adjusted phenotypes from the validation set for each trait. The average predictive ability summarized the 25 iterations. Fisher's tests compared average predictive abilities based on MIRS preprocessing methods.

Figure 1 shows the average predictive abilities when 6 preprocessing data methods were applied to MIRS.mean using the RR-BLUP method. Preprocessing had no significant impact on predictive ability estimates (*P*-values >0.90) compared with the absence of pretreatment, with differences between methods ranging from 0.01 to 0.07 across traits. The smallest differences in predictive ability were observed for PC ranging from 0.76 with the der2 method to 0.77 with norm_der2, whereas the largest were observed for UC ranging from −0.01 with der1 and 0.06 without preprocessing (raw). Standard deviations of the predictive abilities from 25 iterations were low for all pretreatments and traits, ranging from 0.03 (FY using dt) to 0.07 (LSCS using der2). These results suggested that no method stands out from the others, regardless of the trait considered. Brault et al. (2022) found minimal impact of NIRS preprocessing on predictive abilities in grapevines, and Dallinger et al. (2023) observed minor differences in wheat, with Savitzky–Golay filtering generally being the most effective for some traits. Other studies typically used the first derivative or combined it with normalization (Rincent et al., 2018; Lane et al., 2020; Zhu et al., 2021). Given these nonsignificant differences observed on our data, standardized raw spectra were retained for further analysis.

**Figure 1:**
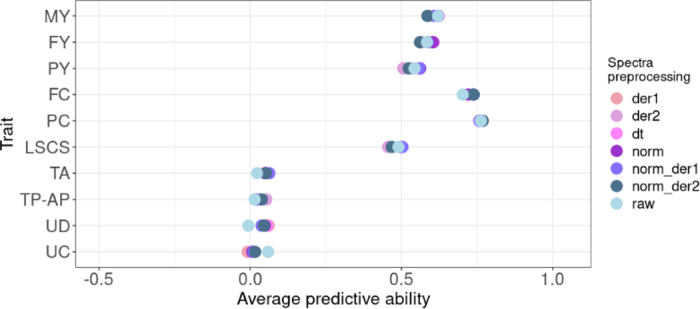
Average predictive ability of milk MIRS.mean with RR-BLUP method for milk production and functional traits according to preprocessing MIR spectra methods. MY = milk yield; FY = fat yield; PY = protein yield; FC = fat content; PC = protein content; LSCS = lactation SCS; TA = teat angle; TP-AP = teat position anteroposterior; UD = udder depth; UC = udder cleft; norm = normalization on standardized raw; dt = detrend on standardized raw; der1 = first derivative on standardized raw spectra; der2 = second derivative on standardized raw spectra; norm_der1 = first derivative on normalized spectra; norm_der2 = second derivative on normalized spectra; raw = standardized raw spectra.

Table 1 presented the average predictive abilities with RR-BLUP and Bayesian RKHS methods based on SNPs or raw milk MIRS (mean, at lactation stage 2 or 6) for milk production and functional traits. The average genomic predictive ability for MY, PY, FC, and PC was identical with RR-BLUP and RKHS methods using SNP information (0.38, 0.32, 0.30, and 0.43, respectively). Slight differences were observed for FY and LSCS, where the genomic predictive ability using RR-BLUP (0.33 and 0.04, respectively) was slightly lower than Bayesian RKHS (0.34, 0.05) with SNP information. To assess the consistency of these genomic predictive abilities, they were compared with the square root of heritability estimates reported for Lacaune dairy sheep by Barillet (2007). The heritabilities for MY, PY, and FC were moderate (0.32, 0.26, and 0.28, respectively) and strong for FC and PC traits (0.41 and 0.51, respectively). The square root of these values were higher but consistent with the genomic predictive abilities observed for dairy traits. Surprisingly, the genomic predictive ability of LSCS was very low (0.04), whereas the square root of its heritability was 0.39 (Barillet, 2007). For udder morphology traits (TA, TP-AP, UD, UC), SNP-based models showed moderate predictive abilities, similar with RR-BLUP and RKHS methods, with values of 0.31, 0.20, 0.21, and 0.20, respectively, using Bayesian RKHS. Compared with the square roots of heritability estimates reported by Barillet (2007) (0.59 for TA, 0.57 for UC, and 0.51 for UD), the predictive abilities were consistently lower.

**Table 1:** Average (±SD) predictive abilities with RR-BLUP and Bayesian RKHS methods based on SNPs or standardized raw milk MIRS (mean and lactation stages 2 and 6) for milk production and functional traits

Trait^1^	RR-BLUP				Bayesian RKHS			
	SNPs	MIRS.mean	MIRS.stage2	MIRS.stage6	SNPs	MIRS.mean	MIRS.stage2	MIRS.stage6
MY	0.38 ± 0.04	0.62 ± 0.03	0.48 ± 0.06	0.32 ± 0.03	0.38 ± 0.04	0.61 ± 0.03	0.48 ± 0.05	0.29 ± 0.04
FY	0.33 ± 0.05	0.58 ± 0.04	0.48 ± 0.04	0.30 ± 0.05	0.34 ± 0.04	0.57 ± 0.04	0.48 ± 0.06	0.27 ± 0.04
PY	0.32 ± 0.04	0.54 ± 0.04	0.45 ± 0.05	0.22 ± 0.05	0.32 ± 0.05	0.55 ± 0.04	0.43 ± 0.05	0.22 ± 0.05
FC	0.30 ± 0.05	0.70 ± 0.06	0.55 ± 0.07	0.48 ± 0.08	0.30 ± 0.06	0.71 ± 0.06	0.51 ± 0.04	0.47 ± 0.06
PC	0.43 ± 0.04	0.76 ± 0.03	0.64 ± 0.04	0.62 ± 0.03	0.43 ± 0.05	0.76 ± 0.04	0.63 ± 0.04	0.62 ± 0.05
LSCS	0.04 ± 0.04	0.49 ± 0.06	0.32 ± 0.04	0.27 ± 0.06	0.05 ± 0.05	0.48 ± 0.05	0.32 ± 0.07	0.23 ± 0.03
TA	0.30 ± 0.05	0.02 ± 0.05	0.05 ± 0.05	−0.02 ± 0.06	0.31 ± 0.04	0.04 ± 0.04	0.05 ± 0.07	0.02 ± 0.06
TP-AP	0.21 ± 0.05	0.01 ± 0.04	0.03 ± 0.05	0.00 ± 0.07	0.20 ± 0.05	0.01 ± 0.04	0.00 ± 0.05	0.02 ± 0.05
UD	0.20 ± 0.06	−0.01 ± 0.05	0.02 ± 0.06	−0.08 ± 0.06	0.21 ± 0.05	−0.01 ± 0.05	0.05 ± 0.04	−0.03 ± 0.07
UC	0.22 ± 0.03	0.06 ± 0.05	0.07 ± 0.04	−0.01 ± 0.05	0.20 ± 0.05	0.07 ± 0.06	0.08 ± 0.06	0.02 ± 0.05

1 MY = milk yield; FY = fat yield; PY = protein yield; FC = fat content; PC = protein content; LSCS = lactation SCS; TA = teat angle; TP-AP = teat position anteroposterior; UD = udder depth; UC = udder cleft.

With the RR-BLUP method, replacing genomic markers with MIRS.mean data significantly improved the predictive abilities for milk production traits, especially FC and PC, with average values of 0.70 and 0.76, respectively (Table 1). These results showed that traits most related to milk had the highest predictive ability, particularly FC and PC that are measured by MIRS. In addition, strong genomic correlations were observed between milk composition (FC, PC) and MIRS in dairy cattle and sheep (Du et al., 2020; Machefert et al., 2024), explaining the high predictive ability of milk production traits compared with genomic SNP-based model. For MY, FY, and PY, the average predictive abilities with RR-BLUP method using MIRS.mean data (0.48, 0.48, and 0.45, respectively) were consistently higher than with SNPs data (0.38, 0.33, and 0.32, respectively). The trait for which using MIRS data instead of SNPs was most favorable was LSCS, with an average predictive ability of 0.49 using MIRS.mean and close to zero using SNPs (0.04) with the RR-BLUP method. The ability of MIRS to capture genetic and environmental effects, unlike SNPs which capture only genetic information, could explained why MIRS-based models outperformed SNP-based model for LSCS, which is strongly influenced by the environment (Kaskous et al., 2022). The link between SCS and MIR spectra could be indirect, through changes in milk composition during an udder inflammatory episode. Gruber et al. (2023) indicate that some wavenumbers were identified as particularly relevant for the prediction models of clinical mastitis and ketosis in dairy cows. In dairy cattle, Odah et al. (2024) also reported better prediction for low heritable traits using MIRS over SNPs; however, this was the case for clinical mastitis in the Montbéliarde breed, cow fertility in Normande, and heifer fertility for all breeds, but not for SCC. However, in our study, replacing SNPs with MIRS.mean yielded very low predictive abilities for udder type traits, ranging from −0.01 (for UD) to 0.06 (for UC) using the RR-BLUP method. This finding was consistent with Odah et al. (2024), which indicated that phenomic predictions achieved only 42% of the accuracy of genomic predictions for height at the sacrum in dairy cows.

Table 1 also compares the average phenomic predictive abilities for the milk production and functional traits using MIRS.mean, MIRS.stage2, and MIRS.stage6. For milk production traits (MY, FY, PY, FC, and PC), MIRS.mean provided the strongest predictive ability (0.61, 0.57, 0.55, 0.71, and 0.76, respectively), followed by MIRS.stage2 (0.48, 0.48, 0.43, 0.51, and 0.63, respectively), whereas MIRS.stage6 (0.29, 0.27, 0.22, 0.47, and 0.62, respectively) performed less effectively, using the Bayesian RKHS method. For LSCS, MIRS.mean yielded a strong predictive ability (0.48), whereas MIRS.stage2 and MIRS.stage6 showed lower predictive abilities of 0.32 and 0.23 respectively, using the Bayesian RKHS method. Udder traits (TA, TP-AP, UD, and UC) displayed generally low power predictions with near-zero predictive abilities across all MIRS-based models (from −0.03 for UD using MIRS.stage6 to 0.08 for UC using MIRS.stage2, using the Bayesian RKHS method). Milk composition, particularly milk fatty acid profile, evolved between early and late lactation and during feeding changes, reflecting metabolic fluctuations (Nudda et al., 2014). This suggested that MIRS could effectively capture these variations and then enable cow (Frizzarin et al., 2021) or sheep (Molle et al., 2021) milks from different diets to be discriminated. In addition, Du et al. (2020) showed that lactation stage significantly affected most of the wavenumbers in cow milk MIRS data. Thus, our approach of using MIRS from extreme lactation stages (2 and 6) may reduce predictive accuracy for traits assessed over the entire lactation period, as a single stage did not account for the variations present throughout the lactation. Nevertheless, strong genomic correlations between spectra collected at early and late lactation stages (>0.77) indicated no significant genotype–environment interaction (Machefert et al., 2024). Overall, in phenomic model based on raw milk MIRS (mean, at lactation stage 2 or 6), the average predictive abilities with RR-BLUP and Bayesian RKHS methods were equal or very close for all traits. Zhu et al. (2021) reported minimal differences between phenomic models (RR-BLUP and Bayesian models) for seed yield prediction, with predictive abilities varying between 0.73 and 0.76.

Figure 2 presents predictive abilities with Bayesian RKHS methods based on SNPs, raw milk MIRS.mean, and both SNPs and MIRS.mean data for milk production and functional traits. Combining SNPs and MIRS.mean data gave the same predictive ability for udder traits (0.31, 0.19, 0.20, and 0.20 for TA, TP-AP, UD, and UC, respectively) as genomic data alone (0.31, 0.20, 0.31, and 0.20, respectively). Combining SNPs and MIRS.mean data performed best for MY, FY, PY, FC, PC, and LSCS (0.65, 0.61, 0.58, 0.72, 0.77, and 0.49, respectively). However, this association showed only slight improvements over using MIRS.mean alone (0.61, 0.57, 0.58, 0.71, 0.76, and 0.48, respectively). The average predictive abilities improvement with the combined models over the phenomic model (only MIRS.mean) for milk production traits and LSCS was 3.8%, indicating low complementary predictive power of MIRS data by genomic data. Brault et al. (2022) found that integrating NIRS with SNPs in grapevines resulted in marginal improvements (0.40 to 0.41) compared with the use of SNPs alone (0.40). Additionally, Jackson et al. (2023) reported a 6.7% increase in phenomic predictive ability when combining NIRS and SNPs over the NIRS-based model in wheat.

**Figure 2:**
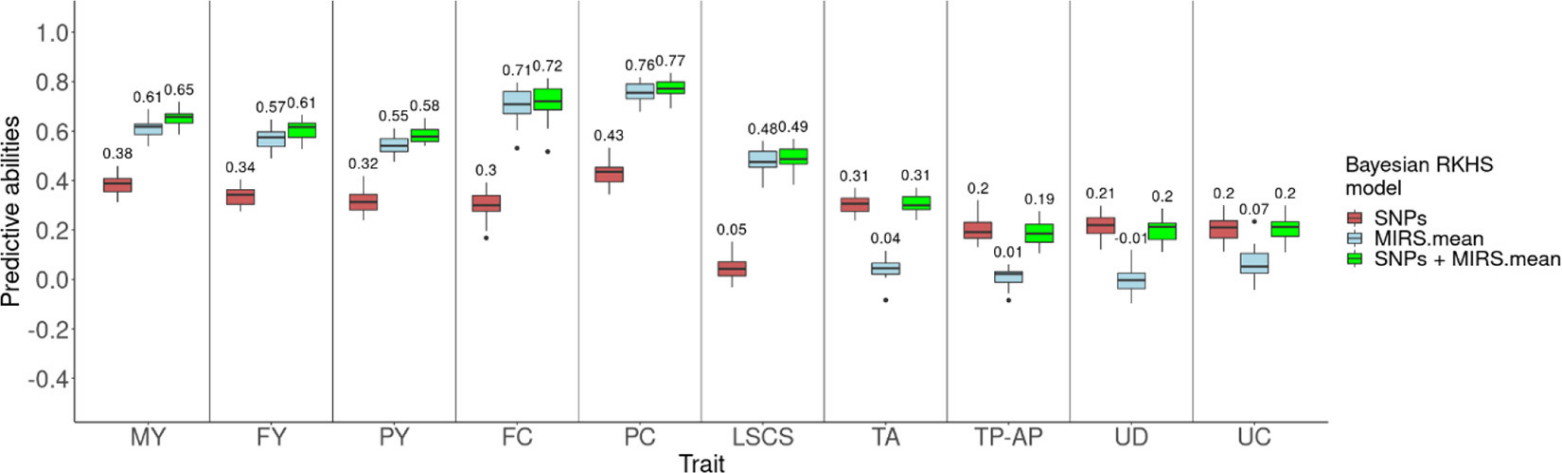
Average predictive ability with Bayesian RKHS methods based on SNPs, standardized raw milk MIRS.mean, or both (SNPs + MIRS.mean) data for milk production and functional traits. The number above the boxplots indicates the average predictive ability per boxplot. In boxplots, boxes indicate the interquartile range (Q1–Q3), midlines the median, whiskers extend to 1.5 times the interquartile range, and dots are outliers. MY = milk yield; FY = fat yield; PY = protein yield; FC = fat content; PC = protein content; LSCS = lactation SCS; TA = teat angle; TP-AP = teat position anteroposterior; UD = udder depth; UC = udder cleft.

In summary, we showed that phenomic prediction performed well for dairy traits, providing better predictive abilities than genomic prediction, but was ineffective for udder morphology traits in Lacaune dairy sheep. The potential of phenomic prediction for more complex or expensive-to-measure traits could be of particular interest but needs further investigations. Furthermore, the combination of MIRS and SNPs data did not markedly enhance the predictive ability of phenomic predictions. In the other hand, the high costs of genotyping limit its use for selecting only females in dairy sheep, but phenomic selection could be used to improve the selection of dams of rams to predict their individual performance with a greater accuracy. Investigating approaches such as single-step GBLUP (Misztal et al., 2013) to infer phenomic values of nonphenotyped individuals for milk MIRS, such as males, using the estimated phenomic values of related females could extend the scope of phenomic selection. Nevertheless, integrating phenomic approaches could be an innovative strategy for improving the overall efficiency of selection in dairy sheep, but further preliminary studies are required into assessing the ability of milk MIRS to predict phenotypes in the next generation before a potential implementation.
